# When memory leads the brain to take scenes at face value: face areas are reactivated at test by scenes that were paired with faces at study

**DOI:** 10.3389/fnhum.2014.00018

**Published:** 2014-01-29

**Authors:** John A. Walker, Kathy A. Low, Neal J. Cohen, Monica Fabiani, Gabriele Gratton

**Affiliations:** ^1^Beckman Institute, University of Illinois at Urbana-ChampaignUrbana, IL, USA; ^2^Psychology Department, University of Illinois at Urbana-ChampaignChampaign, IL, USA

**Keywords:** relational memory, cortical reactivation, face processing, scene processing, brain imaging, event-related optical signal (EROS)

## Abstract

In the first use of the event-related optical signal as a brain imaging tool for the study of long-term memory, we examined relational or associative aspects of memory, widely presumed to involve the interplay among multiple brain regions in representing and reactivating different elements of a given event. Here, we found that a brain region known to be involved in face processing (the posterior superior temporal sulcus) was active not only when viewing faces during the study phase but also when viewing scenes at test that, through prior learning, were associated with specific faces. These findings, demonstrating the activation of stimulus-specific cortical regions in the absence of stimuli of that type, based on learned relations, reveal cortical substrates of the reactivation of relational memories.

## WHEN MEMORY LEADS THE BRAIN TO TAKE SCENES AT FACE VALUE

For decades we have known that memories for complex events are not located in one place in the brain but rather distributed across multiple sites in the cortex, with different elements of the event stored in different cortical processors (Lashley, 1950; Norman and O’Reilly, 2003). Memories seem to be tied heavily to the particular processing operations, and to be stored in the specific processing regions, engaged when a person initially experiences the event (Marr, 1971; Norman and O’Reilly, 2003; Hofstetter et al., 2012). For example, when asked to remember a place, participants activate areas of the brain very similar to those that were active when first viewing that place (O’Craven and Kanwisher, 2000), in both cases involving the “parahippocampal place area.” Similarly, the “fusiform face area,” strongly associated with the processing of faces (Kanwisher et al., 1997), is also activated when participants recall and imagine faces (O’Craven and Kanwisher, 2000). More generally, during remembering participants activate those sensory processing areas of the brain that were specifically associated with particular types of to-be-remembered items during their initial processing. This is known to be the case not only for faces or places, but also for colors (Simmons et al., 2007), tools (Chao et al., 2002), words (Hofstetter et al., 2012), and others.

This intimate connection of information processing with memory storage has been best documented for individual stimuli, as in the examples above. However, most real-world events are complex, involving multiple items and sensory modalities. Therefore memory for any particular event would necessarily involve the corresponding cortical processors engaged during initial learning. Similarly, retrieval of the event would require the orchestrated reactivation of these different cortical regions. This reactivation could be prompted by the presentation of a single element of the event, recruiting back the entire assembly of cortical areas that were involved in its initial processing. Furthermore the element of an event that prompts this reactivation does not necessarily have to be a semantically related associate such as those used in the experiments above, but could also be otherwise independent items, related to one-another through a single episode. Although postulated from a theoretical standpoint, this episodic “relational reactivation” has not been yet demonstrated.

Some theoretical work has pointed to the relational or associative nature of memory for events (Marr, 1971; Morris et al., 1977; Cohen and Eichenbaum, 1993; Eichenbaum and Cohen, 2001; Norman and O’Reilly, 2003; Mayes et al., 2007), emphasizing distributed representations of the links among the constituent elements of the event, with a critical mediating role played by the hippocampus both in the initial binding of the relations in memory and in their later retrieval or reactivation (Eichenbaum, 2000; Norman and O’Reilly, 2003; Mayes et al., 2007; Hofstetter et al., 2012). A considerable body of findings in humans has provided evidence about the hippocampus’s unique ability to create and flexibly use bindings of arbitrary or otherwise unassociated items after just one exposure (Cohen and Eichenbaum, 1993; Ryan et al., 2000; Eichenbaum and Cohen, 2001; Hannula et al., 2006, 2007; Konkel et al., 2008; Hannula and Ranganath, 2009). However, there is little evidence about the cortical interactions in the reactivation of linked elements (relational memories), that is, about the ability of previously acquired associations or relations among items to cause cortical representations in one region to reactivate related representations elsewhere in cortex. The relational memory theory would predict that the hippocampus can use the binding between elements to reactivate one element in the absence of the other (Cohen and Eichenbaum, 1993; Eichenbaum, 2000; Eichenbaum and Cohen, 2001). Previous studies have shown stimulus-specific activity when shown a relational cue. However these studies have either had the participant actively imagine the to be recalled item (Johnson and Rugg, 2007; Staresina et al., 2012; Staresina et al., 2013; For interaction of imageability and paired associate recall see Madan et al., 2010) or displayed the item on screen (Hofstetter et al., 2012). With paradigms such as these it is difficult to tell if the observed activity is due to reactivation elicited by the relational cue or due to the presence of the item, either from the displayed item or through mental imagery. One recent study used multi-voxel pattern analysis to classify patterns of activation and reactivation (Zeithamova et al., 2012), showing that large-scale brain activation patterns match between study and reactivation. However this technique does not offer the means to say anything about the participation of the various cortical regions representing different elements of the relational memory of the event – what might be called relational reactivation.

The current study endeavored to document processes that contribute to relational reactivation. By relational reactivation we mean *specific* cortical activity indexing processing of an item elicited by the presentation of another item previously associated with it during a prior learning episode. To this end we employed a modified version of a paradigm successfully used in previous investigations of relational memory (e.g., Hannula et al., 2007; Hannula and Ranganath, 2009). Participants studied a series of arbitrary face-scene pairings and later performed a recognition memory test for those pairs. A critical feature of this paradigm is “scene preview” – the presentation of an individual scene from a previously studied pairing prior to each scene-face test display. Previewing the scene makes it possible for the participant to reactivate the associated face *prior *to the presentation of the combined face-scene test display. Previous work with an eye-tracking version of this paradigm took advantage of this opportunity for reactivation, showing that having a scene preview produced disproportionate early viewing of the associated face in the scene-face test display (greater and earlier viewing of the associated face relative to other equally familiar faces when there was a scene preview compared to when the scenes and faces were presented simultaneously; Hannula et al., 2007). This effect was shown to be associated with increased hippocampal activation during the scene preview period, which predicted subsequent memory performance (Hannula and Ranganath, 2009), and was absent in patients with amnesia following hippocampal damage, demonstrating the critical role of hippocampus in its elicitation (Hannula et al., 2007).

In the current study we focused on the cortical components of reactivation and on their temporal dynamics, and examined brain activity in cortical regions engaged by face processing elicited by the presentation of ***scenes*** that were associated with those faces during previous learning. We compared brain activity occurring during the scene preview period for scenes that were associated to specific faces during the previous encoding period with the activity elicited by scenes that were presented for the first time at test. We interpreted activity observed in face-related areas during the viewing of scenes previously associated with faces and *not* observed during the viewing of scenes that were not paired with faces as an index that reactivation of the associated face representations may be occurring. We then compared this “reactivation-related” activity with that elicited by the first presentation of faces at encoding time (i.e., when they were yet to be associated with any scene), examining both their spatial and temporal characteristics.

To accomplish this we utilized a technology that has good temporal and spatial resolution, the event-related optical signal (EROS; Gratton and Fabiani, 2010). EROS measures changes in the optical scattering properties of the cortex as a result of neuronal activity, by observing the amount of time it takes for near-infrared light to pass through the cortex. As the technology measures neuronal activity instead of the hemodynamic signals imaged by functional magnetic resonance imaging (fMRI), EROS has a temporal resolution on the order of milliseconds, similar to that of electroencephalography (EEG), while achieving a spatial resolution on the order of centimeters, similar to that of fMRI. This combination of temporal and spatial resolution enables EROS to determine not only where activity is taking place but also when the activity is occurring. This allows the user to investigate the order of activation of various brain areas, not just that those areas were active during a given trial. EROS is limited in its depth-sensitivity. However, previous studies have shown that EROS is sensitive to activity as deep as about 3.0 cm from the head surface (Gratton et al., 2000), making it suitable for assessing activations and reactivations in most cortical areas. This paper presents the first use of this technique to study the spatial and temporal dynamics of episodic memory reactivation.

**FIGURE 1:**
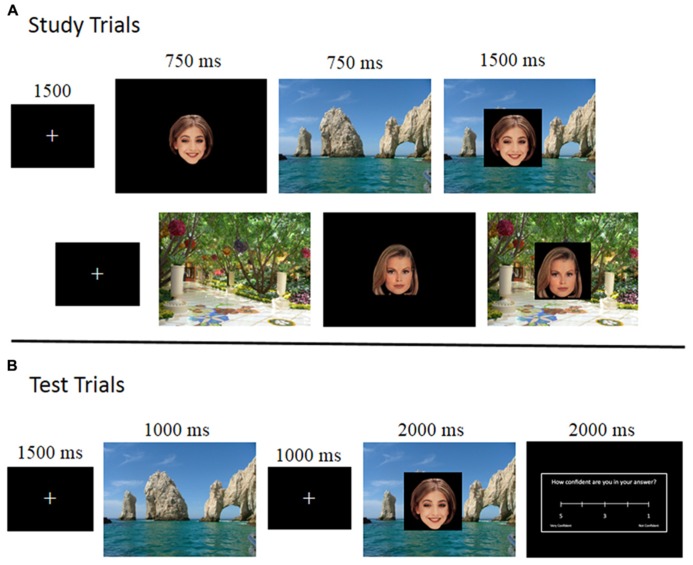
**Schematic representation of the experimental paradigm, illustrating the sequence of stimuli during study (A), where the top row represents a face-first trial and the second row represent a scene-first trial, and test (B).** Note that, during test, scenes were always presented first, generating a **scene preview period**.

Participants studied pairs of faces and scenes, first viewing either a face or a scene individually followed by the pair together (see **Figure 1**). At test, participants were given an old/new recognition test for each of the face-scene pairs, each preceded by a *scene preview*. We found scene-elicited activity during scene previews in the posterior superior temporal sulcus (STS), an area easily accessible by the EROS technique. Critically, (1) the activity was greater for “old” scene previews compared to “novel” scene previews (which have no face associated with them), (2) the activity was elicited in the *same region that was activated by faces presented alone* during the study phase (localizer), and (3) STS is an area known to be part of the network of areas involved in processing faces, being shown to be active during both general face processing (Puce et al., 1998, 2003; Grill-Spector et al., 2004; Fairhall and Ishai, 2007) as well as social judgments about a face (Puce et al., 1998; Hoffman and Haxby, 2000). We interpret this STS activation as a manifestation of the reactivation of relational memory about the face that, through prior episodic learning, was associated with the presented scene. The reactivation was observed at two different temporal intervals: at a short latency (only 100 ms later than the activity elicited by faces alone during initial study) and a longer latency, which was associated with dorsolateral prefrontal cortex (DLPFC) activity, a region of the cortex implicated in top-down control and relational memory retrieval during recall and recognition (Ranganath et al., 2000; Duzel et al., 2004; Hannula and Ranganath, 2009).

## MATERIALS AND METHODS

### PARTICIPANTS

Twenty-one right-handed young adults (10 women; mean age = 23.38 SD = 4.19) participated in this study for a payment of $15 an hour. Three participants were excluded from the analysis due to withdrawal from the experiment prior to completion. All participants indicated that they had normal or corrected to normal vision and were not taking medications that would affect the central nervous system. Informed consent was obtained from each participant and all procedures were approved by the University of Illinois Institutional Review Board.

### STIMULI

The stimuli consisted of 588 full-color face images (294 female faces) selected from a previously normed faces database (Althoff and Cohen, 1999) and 882 scenes from Brand X© photography. The faces were all sized to 300 × 300 pixels and the scenes were all sized to be 800 × 600 pixels, filling the entirety of the screen.

### PROCEDURES

Following informed consent, each participant was fit with an EROS recording helmet (see below) and were given a practice block of 12 study trials followed by 12 test trials so that she/he could get used to the timing of the trials. Following the practice block the participant did eight study/test blocks across two days with four blocks each day. The time between the first and second day was anywhere between 1 and 7 days with an average of 4.2 days. Participants were allowed to take breaks in between each block as necessary and were given the option to retake the same practice block at the beginning of the second day.

#### Study block

Study blocks consisted of 72 study trials divided into two sets of 36 trials. Each set of study trials started with a 1 s fixation cross. As can be seen in **Figure 1A**, the study trials started with a face and scene being shown individually for 750 ms each. Half the study trials had the face shown first (“face-first” trials) and half the trials had the scene shown first (“scene-first” trials). After the face and scene were shown individually, the face was superimposed on the center of the scene for 1500 ms. Participants were instructed to study each of the pairings as they were to be tested on those pairings later on. The blocks were divided by short breaks to minimize movement artifacts.

#### Test block

Following each study block was a corresponding test block of 72 test trials, testing the pairs of items studied in the immediately preceding study block. These test trials were divided up into three sets of 24 test trials, with each set beginning with a 1 s fixation cross. As can be seen in **Figure 1B**, a test trial consisted of a scene being presented for 1000 ms (the scene preview), followed by a fixation cross for 1000 ms, followed by the face superimposed on the center of the scene for 2000 ms. Participants were instructed to respond using a button box as to whether or not a face-scene pair was studied together during the previous study block (old–new judgment). There were three types of test trials: match, re-pair, and novel. Match test trials were test trials in which the face and the scene being tested had been presented together during the study phase. The re-pair test trials were comprised of faces and scenes that had been presented in the study phase, but had not been paired together. The novel test trials were comprised of a novel scene with a previously studied face. The correct response for match trials was “old” as those pairs were previously studied together, whereas the correct response for re-pair and novel trials was “new” as the pairs in those trials were not studied together. Participants were asked to respond only once the face had appeared, and were explicitly told not to respond during the scene preview or fixation.

Following the scene and face combination the participants were allowed 2000 ms to provide a confidence rating on their answer using a 5-point Likert scale (with 1 being not confident and 5 being very confident). Every test trial ended in a fixation for 1500 ms.

Counterbalancing for the study and test blocks consisted of four lists of 576 face-scene pairings created by randomly pairing 576 of the faces and 576 of the scenes and then randomly assigning each pair to each study and test type condition. Each pair was then randomly assigned to one of eight blocks of 72 items each with the stipulation that each block contains 12 of each study-test type combination [(face first, scene first) × (match, re-pair, novel)]. Of the remaining scenes, 288 were then randomly assigned to replace the scenes in the novel test trials. Four different lists were created to ensure that every scene was tested in one of the three categories. No scene and face were paired together more than once across these lists. The remaining 12 faces and 18 scenes were used to create a practice block of 12 study trials followed by 12 test trials. These faces and scenes were not included in the four lists and the practice trials were the same for all participants.

### OPTICAL RECORDING

Optical data were recorded over 2 days using six synchronized ISS model 96208 frequency domain oxymeters (Imagent®; ISS, Inc., Champaign, IL, USA). The light sources were laser diodes emitting light at the wavelength of 830 nm (max amplitude: 10 mW, mean amplitude after multiplexing: 1 mW) modulated at 110 MHz. Optic fibers were used to channel each light to the surface of the scalp. The detectors were fiber optic bundles (diameter = 3 mm) connected to photomultiplier tubes (PMTs). The PMTs were fed with a current modulated at 110.0625 kHz, generating a heterodyning frequency of 6.25 kHz. The output current from the PMTs was digitized at 50 kHz, affording 8 points per heterodyning cycle. A time-multiplexing approach was used to record from sixteen sources for each detector. In this approach, each source was switched on for 1.6 ms, and off for 24 ms. This allowed to record for a total of 10 heterodyning cycles (80 points) for each multiplexing time unit. However, to avoid cross-talk, the first two cycles were discarded, and the remaining 64 points were subjected to a fast Fourier transform for computation of DC (average) intensity, AC (amplitude), and relative phase delay (in degrees and later converted to picoseconds). Only phase delay data are reported here.

**FIGURE 2:**
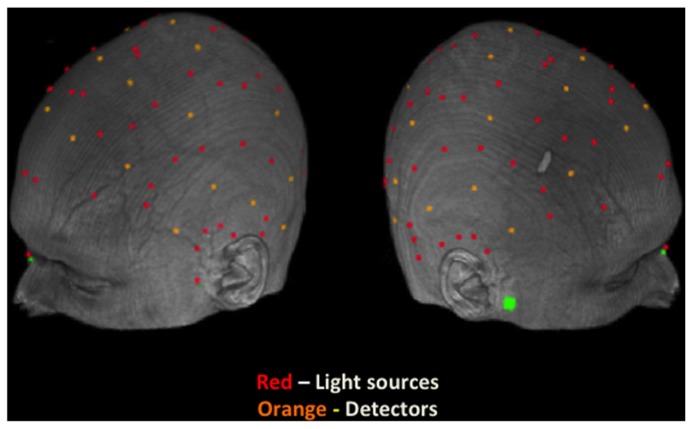
**Locations of sources and detectors on the scalp of a representative participant.** Light sources are indicated by a red dot and detectors are indicated by an orange dot. Green marks denote fiducial points (nasion and preauricular locations).

**FIGURE 3:**
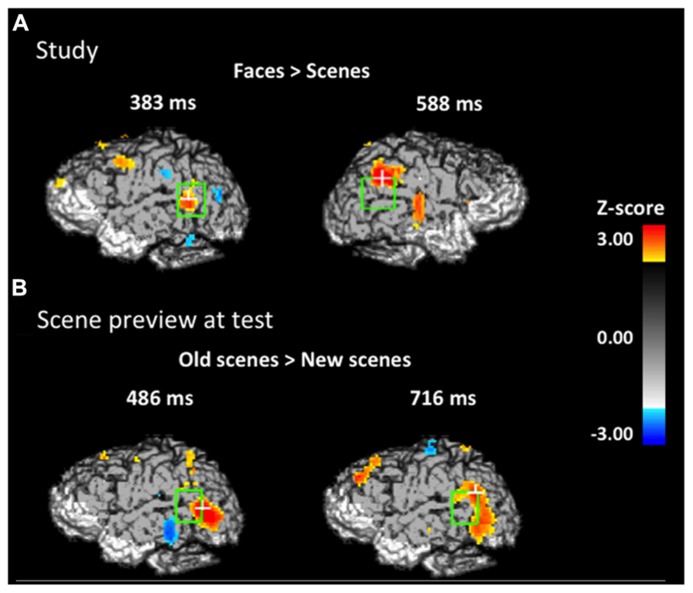
**Spatial maps based on group-level *Z*-statistics of the EROS data projected on sagittal brain surfaces.** Dark gray shading represents the brain area sampled by the recording montage. The light green rectangle indicates the STS ROI and the white cross marks the peak resel within the ROI. **(A)** Activity during study trials for face-first trials versus scene-first trials in the left STS at 383 ms and in the right STS at 588 ms. **(B)** Activity during the scene preview for previously studied scenes versus completely novel scenes in the left STS at 486 ms and 716 ms.

**FIGURE 4:**
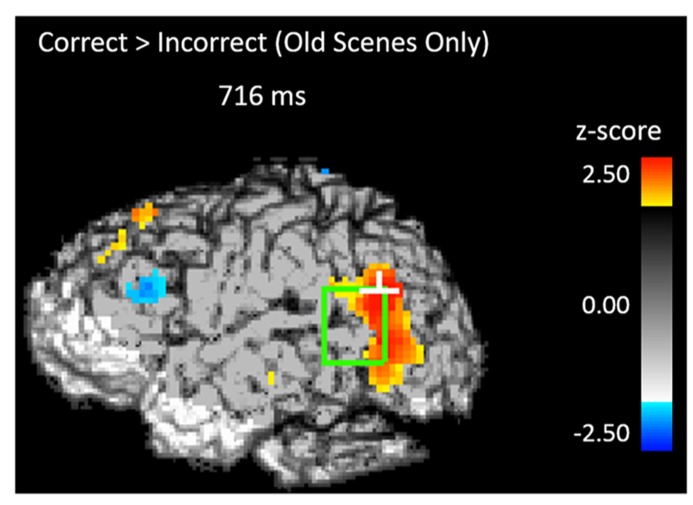
**Activity during the scene preview for subsequently correct trials vs. subsequently incorrect trials in the left STS at 716 ms.** The light green rectangle indicates the STS ROI and the white cross marks the peak resel within the ROI.

**FIGURE 5:**
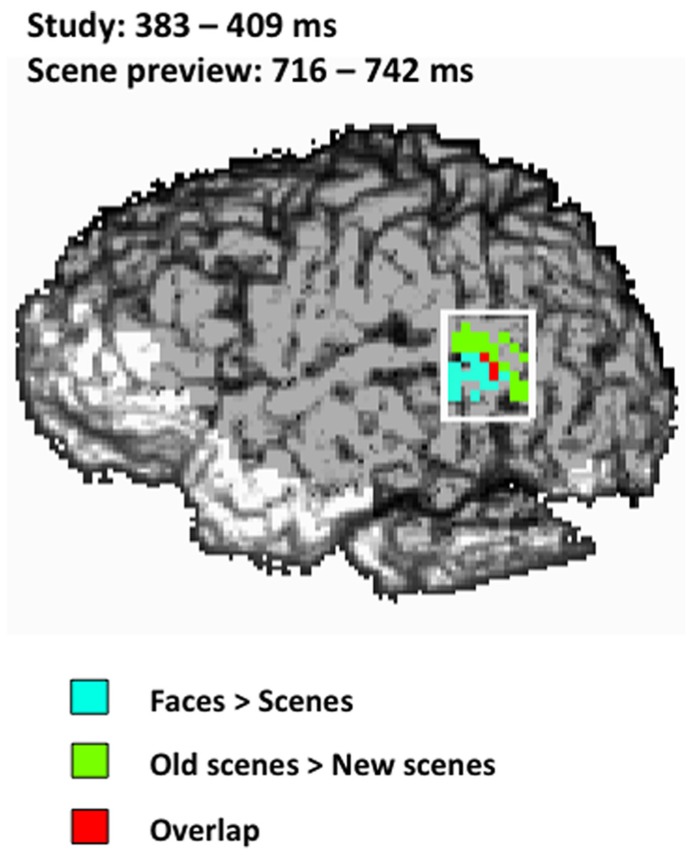
**Spatial map of the conjunction analysis projected onto the left sagittal surface at a threshold of *p *< 0.04 for the conjunction (*p *< 0.2 for each condition separately).** Blue represents the activity for faces > scenes from 383–409 ms during study, green represents the activity for old scenes > new scenes from 716–742 ms during the scene preview, and red represents the overlap between those two activations. The white rectangle indicates the STS ROI.

**FIGURE 6:**
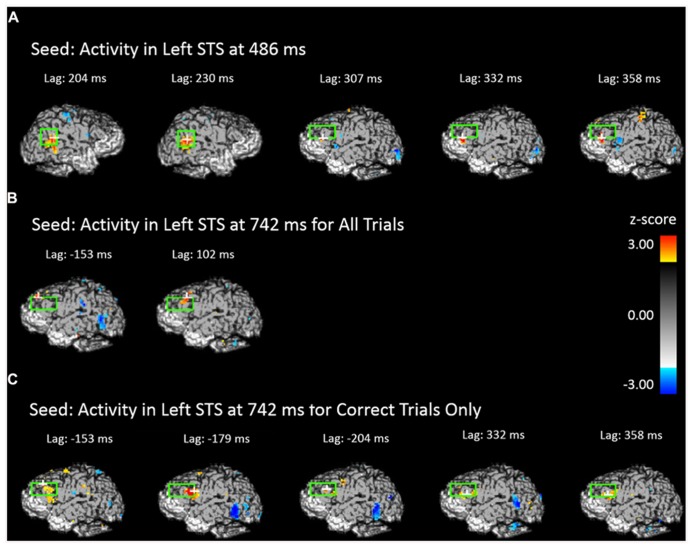
**Cross-correlational analyses using the peak activity during the scene preview for the contrast of old scenes > new scenes as the seed resel.** The light green rectangle indicates the STS ROI for the first two images and the DLPFC ROI for all others. The white cross marks the peak resel within the ROI. **(A)** Areas of the brain and time shift that show above-threshold correlation in activity with the peak resel during the scene preview at 486 ms. **(B)** Areas of the brain and time shift that show above-threshold correlation in activity with the peak resel during the scene preview at 742 ms for all trials. **(C)** Areas of the brain and time shift that show above-threshold correlation in activity with the peak resel during the scene preview at 742 ms for correct trials only.

Source and detector fibers were mounted on a modified motorcycle helmet. The area covered by our montage covers the entirety of the top of the head. The source and detector locations can be seen in **Figure 2** and the coverage of the montage can be seen in **Figures 3–6**, represented by the darker gray shading on the brain. Our montage consisted of 24 detectors and 64 sources. Source-detector distances ranged between 15 and 102 mm. To avoid cross-talk, the sources were arranged such that during any given time division of the multiplexing cycle only one source was within 6 cm of any given detector. This allowed us to record from 384 channels (pairings of source and detector) at 39.0625 Hz on each of the 2 days, for a total of 768 channels.

The locations of the sources and detectors were digitized with a Polhemus “3Space”® (Colchester, VT) 3D digitizer and co-registered with a volumetric T1-weighted MR image for each subject (Whalen et al., 2008). The co-registered data were then Talairach-transformed to permit registration across subjects. The phase data were corrected off-line for phase wrapping, pulse artifacts were removed (Gratton and Corballis, 1995), and the data were low-pass filtered to 5 Hz (Maclin et al., 2003). Channels with standard deviations of the phase greater than 100 ps were excluded from further analysis (for further details of these analytic steps, see (Gratton and Fabiani, 2007).

### OPTICAL STATISTICAL ANALYSES

The phase data were divided into epochs around stimulus events of interest with 204.8 ms pre-stimulus baseline and 768 ms post-stimulus recording for the study phase. The time locking event of interest was the onset of the first stimulus of a study trial (either the face in trials where the face was presented first or the scene in trials when the scene was presented first). For the test phase the phase data were also divided into epochs around the stimulus events of interest with a 204.8 ms baseline but the post-stimulus recording consisted of 2022 ms so that it could include the scene preview and the fixation cross, both shown before the face is presented in the test trial.

In-house software “OPT-3D” (Gratton, 2000) was used to reconstruct the optical path for each channel spatially, combine channels whose mean diffusion paths intersected for a given brain volume (voxel) and to compute group-level statistics. The resel size of the cortical projections were determined by the independence of the error terms at various voxel distances computed by using the methods described by Worsley et al. (1999). An 8 mm Gaussian filter (based on a 2 cm kernel) was used to spatially filter the data. The group-level statistics were then converted to *Z*-scores and compared to critical *Z*-scores based on the number of resels within an region of interest (ROI) and the subsequent correction for multiple comparisons. These *Z*-scores are then orthogonally projected onto images of the sagittal surfaces of the brain in Talairach space (Talairach and Tournoux, 1988).

Due to its high spatial and temporal resolution (which may inflate the number of comparisons), statistical analysis of EROS during both the study and test phase was limited to a priori ROIs. Whole-brain analyses, as are often done in fMRI, which has only high spatial resolution, are not practical with EROS data as the number of data points (one for every resel at every time point) would make the correction for multiple comparisons too severe. Thus we focused on the STS and the DLPFC, areas that have been shown to be important in the processing of faces (Puce et al., 1998, 2003; Grill-Spector et al., 2004; Fairhall and Ishai, 2007) and in the top-down control of memory retrieval (Miller and Cohen, 2001), respectively. These areas are also easily accessible with optical imaging, whereas other potential areas of interest such as the fusiform gyrus and the ventrolateral prefrontal cortex could not be accessed with the instrumentation used in the current study (For the spatial extent of areas covered see **Figures 3–6**). The boundaries for the STS ROI were defined in Talairach space (Talairach and Tournoux, 1988) as *y = -*65 to *-*43, and *z *= *-*8 to 20 and *y = -*72 to *-*45, and *z *= *-*3 to 24 for the left and right STS, respectively. These boundaries were based on the peak activation in the STS for faces in previous fMRI work (Bonda et al., 1996; Kanwisher et al., 1997; Puce et al., 1998, 2003; Haxby et al., 1999; Hoffman and Haxby, 2000; Ishai et al., 2000, 2005; Grill-Spector et al., 2004; Fairhall and Ishai, 2007). The boundaries for the DLPFC ROI were defined as *y = *10 to 50, and *z *= 15 to 35 for both the left and right DLPFC. These boundaries were based on the spatial extent of Brodmann’s areas 9 and 46.

Additionally, in order to control for multiple comparisons we also limited our analyses to temporal intervals of interest (IOIs) at both study and test. Previous work using intracranial event-related potentials has shown activity in the STS to the presentation of faces at around 170 ms, between 200 and 650 ms and around 700 ms (Allison et al., 1999), therefore for our analyses at study we used an IOI of 150 to 750 ms. As no one has yet examined the timing of STS activity during reactivation of faces, we had to rely on other techniques with similar temporal resolution in order to create our IOIs. Previous work has shown that eyes disproportionately start to fixate on the matching face in the time range of 500–1500 ms in a similar paradigm if more than one face is present (Hannula et al., 2007). We limited our scene preview (i.e., reactivation) analyses to this time frame. However, for exploratory reasons we also report any activity that was found to be significant with a spatial correction in **Table 2**. Correction for multiple comparisons across voxels was applied based on the number of independent resolution elements (resels) within each ROI using random field theory (Friston et al., 1995; Gratton, 2000; Maclin et al., 2003).

## RESULTS

### BEHAVIORAL RESULTS AT TEST DISPLAY

**Table 1** provides accuracy and reaction times for each condition. Overall participants made correct old–new judgments on 74% (*M = *0*.*74, SD = 0.09) of the trials with an average response time of 1076.18 ms (SD = 181.75). Not surprisingly given that in 2/3 of the trials “new” was the correct response, participants were more likely to indicate that a pair of items was a new pair (“new” response; *M = *0*.*71; SD = 0.06) than they were to respond that a pair was an old pair, *t*(15) = 13.554, *p *< 0.001, indicating a bias to respond “new”. However, their overall performance on the task was still above chance for all items as indicated by the d’ calculations, which take bias into account (*M* = 1.21, SD = 0.68, *t*(15) = 7.092, *p *< 0.001). This was the case even when only old and re-pair trials (i.e., those trials in which all items were equally familiar) were considered to calculate d’ (*M *= 1.0, SD = 0.71), *t*(15) = 5.636, *p *< 0.001. Furthermore, participants’ performance was still above chance in all three test trial types (match, re-pair, and novel), all *p* values < 0.05.

**Table 1:** Proportion of correct recognition responses and response times for each trial type.

	Response accuracy		Response time (ms)	
Test trial type	*M*	*SD*	*M*	*SD*
Match	0.55	0.15	1209	218
Re-pair	0.78	0.11	1116	178
Novel	0.89	0.07	904	190

Additionally, there was an overall effect of test trial type on both accuracy [*F*(2,30) = 56.49, *p *< 0.001], and response time [*F*(2,30) = 47.69, *p *< 0.001]. Bonferroni-corrected pair-wise *t* tests showed that participants were less accurate on match/“old” trials (*M* = 0.55; SD = 0.15) than on either novel (*M = *0.89; SD = 0.08;* t*(15) = -9.80, *p *< 0.001), or re-pair trials (*M = *0*.*78; SD = 0.11; *t*(15) = 5.76, *p *< 0.001), both of which required a “new” response. Participants were also more accurate on novel than re-pair trials [*t*(15) = 5.50, *p < *0.001]. In terms of reaction time, participants were slower to respond during match trials (*M* = 1208.74 ms; SD = 220.17 ms) than either novel (*M = *903.85 ms; SD = 190.19 ms; *t*(12) = -7.65, *p* < 0.001; *t*(15) = -8.98, *p *< 0.001) or re-pair trials (*M* = 1115.96 ms; SD = 178.02 ms;* t*(15) = -3.05, *p* = <0.01). They were also slower in responding to re-pair trials than to novel trials, *t*(15) = -8.98, *p *< 0.001. The increase in reaction time and the decrease in accuracy for re-pair compared to novel trials could indicate the presence of some level of conflict during the re-pair trials. This conflict could be due to reactivation (since a face could have been reactivated by the scene preview that did not match the face being tested). Conflict could also be the result of discrepancies between item memory (since the scene and face are both “old” on re-pair trials) and relational memory (as the two old items were not studied together).

### ANALYSIS OF EROS RESULTS

#### Activity at study

In order to establish that any reactivation specific to the retrieval of studied faces took place at test when the associated scenes were presented, we had to first establish what activity was uniquely elicited by faces (and not by scenes) during study (localizer task). To identify this activity, we took advantage of the fact that we presented two different types of trials at study, those for which a face was presented first (face-first trials) and those for which a scene was presented first (scene-first trials). This allowed us to determine which brain activity is specific to face-first trials, by subtracting from it the activity elicited by scene-first trials. Note that this contrast takes advantage of the temporal resolution of EROS, which allowed us to examine the activity elicited by the first stimulus in the study pair (face or scene) in the absence of any activity elicited by the presentation of the second stimulus in the pair. **Figure 3A** shows the statistical maps of EROS data for the contrast of *faces (first) > scenes* (*first)* at study. As expected, this contrast revealed significant preferential activation for faces over scenes in both left and right STS. The preferential activation for faces was significant in the left STS between 383–409 ms after stimulus onset (peak *Z* = 2.636, *Z_crit_* = 2.5; Talairach coordinates: *y* = -53, *z = *4) and in the right STS from 588–614 ms (peak *Z* = 2.675, *Z_crit_* = 2.62). Subsequent analyses revealed that these differential effects were due to activation (compared to a pre-stimulus baseline) in the face-first condition, and not solely to a significant negative deviation from baseline for the scene-first condition. At no point during study did the STS show activation levels for scenes that were greater than baseline values.

#### Activity at test during scene preview

If participants are using the scene preview to reactivate the associated face in preparation for the upcoming test display, there should be activation at test in face-specific areas (i.e., STS) in response to those scenes that had faces associated with them, compared to novel scenes. To test this hypothesis, we compared test trials including a previously studied scene (match and re-pair trials) to the trials that included a scene that was completely novel (novel trials). Specifically we predicted that match and repair trials would elicit activity in the STS at a similar location and latency as the preferential face activation observed during study. **Figure 3B** shows the peak activations in each significant time interval. Supporting the notion that participants were reactivating face areas during the preview of old scenes, there was significant activation in the left STS from 488–511 ms after scene preview onset (peak *Z* = 2.387, *Z_crit_* = 2.32; Talairach coordinates: *y* = -63, *z = *7). Note that this latency was only 100 ms longer than that for activity found during the study phase for faces (383 ms). Interestingly, we also found significant activation in the left STS at a later time window from 716–742 ms (peak *Z* = 3.391, *Z_crit_* = 2.73; Talairach coordinates: *y* = -61, *z = *-1). In the right STS there was also a marginally significant activation for old scene previews at 1254 ms (peak *Z* = 2.566, *Z_crit_* = 2.71; Talairach coordinates: *y* = -46, *z = *-3). We also examined the subset of trials in which the participant correctly responded to the subsequent old/new judgment. For these correct trials, only the time period from 716–742 ms showed reliable activation of the left STS (peak *Z* = 2.925, *Z_crit_* = 2.71). To see if this activity is related to behavior, we contrasted correct trials versus incorrect trials for previously studied scenes and found greater activity in the left STS for correct trials during this same time period (See **Figure 4**; peak *Z *= 2.754, *Z_crit_* = 2.64) The same contrast between correct and incorrect trials during the earlier time period (488–511 ms) was non-significant (peak *Z = *0.743, *Z_crit_* = 2.36).

To determine the amount of overlap between the activity for faces seen during study and the activity for old scene previews at test in the left STS we performed a conjunction analysis (Price and Friston, 1997) for both the early and late activations during the scene preview. There was no overlap between study activation and scene preview activation in the early window (486 ms); however we did find overlap with the later scene preview activation (716–742 ms) at a combined threshold of *p *< 0.04. As can be seen in **Figure 5**, the overlap between the two activations takes place toward the center of the left STS ROI.

Additionally, we examined the DLPFC (BA 9, 46) as this region is well known to be involved in top-down or controlled processing (Miller and Cohen, 2001) and it is an area that has been shown to be active during successful retrieval of relational pairs (Hannula and Ranganath, 2009). We found significant activation in the left DLPFC for old scenes greater than new scenes early on during the scene preview around 307 ms (peak *Z* = 2.609, *Z_crit_* = 2.59; Talairach coordinates: *y* = 47, *z = *34) and later on during the scene preview from 1100–1126 ms (peak *Z* = 3.711, *Z_crit_* = 2.84; Talairach coordinates: *y* = 32, *z = *34), 1128–1331 ms (peak *Z* = 3.459, *Z_crit_* = 2.72; Talairach coordinates: *y* = 37, *z = *32), and 1663–1715 ms (peak *Z* = 2.992, *Z_crit_* = 2.77; Talairach coordinates: *y* = 37, *z = *34). Furthermore, we also found greater activity for old scenes in the right DLPFC around 281 ms (peak *Z* = 2.978, *Z_crit_* = 2.97; Talairach coordinates: *y* = 22, *z = *24) and 1279 ms (peak *Z* = 2.848, *Z_crit_* = 2.8; Talairach coordinates: *y* = 34, *z = *32). These data are summarized in **Table 2**.

**Table 2:** Locations and statistical analyses for areas found to be active during study (faces > scenes) and scene preview (old scenes > new scenes)

Location	Time period (ms)	Location (*y,z*)	*Z*_obs_	*Z*_crit_
**Study**
Left STS	383–409	(-53, 4)	2.636	2.50
Right STS	588–614	(-58, 23)	2.674	2.57
	691	(-58, 17)	2.446	2.53
**Scene preview – correct trial only**
Left STS	716–742	(-63, -1)	2.925	2.71
Left DLPFC	307	(47, 34)	2.729	2.61
	1254–1305	(37, 34)	2.926	2.73
	1459–1484	(49, 32)	2.872	2.66
	1663–1715	(37, 34)	2.875	2.79
Right DLPFC	1305	(34, 34)	2.665	2.63
	1561	(42, 34)	3.026	2.91
**Scene preview – all trails**
Left STS	486–511	(-63, 7)	2.387	2.32
	716–742	(-61, -1)	3.391	2.73
	1254	(-46, -3)	2.566	2.71
Left DLPFC	307	(47, 34)	2.609	2.59
	1100–1126	(32, 34)	3.711	2.84
	1128–1331	(37, 32)	3.459	2.72
	1663–1715	(37, 34)	2.992	2.77
Right DLPFC	281	(22, 24)	2.978	2.97
	1279	(34, 32)	2.848	2.80

#### Functional connectivity (cross-correlational) analyses

The results presented in the previous section show two periods in which face-related areas are reactivated by previewing old scenes. Before and after these two intervals we also observed significant prefrontal activation, suggesting an involvement of the prefrontal cortex in retrieval-related processes. To elucidate the possible connections between the activity observed in the prefrontal cortex and that observed in the STS we performed forward- and backward-lagged cross-correlation analyses using the peak voxel in the STS in each significant time period as seed points (one from each significant time period in the analysis including all trials and one from the significant time period for correct trials only). For the early (486 ms) all-trials seed, we found a significant correlation between activity in the seed point and later activity in the left DLPFC after a lag of 307 ms (peak *Z* = 3.765, *Z_crit_* = 3.28) and marginally significant correlations at lags of 332 ms (peak *Z* = 2.724, *Z_crit_* = 3.23) and 358 ms (peak *Z* = 3.019, *Z_crit_* = 3.05; **Figure 6A**). In contrast, this early STS seed activity did not correlate significantly with any later lags in the left STS (peak Z = 2.26) even when the alpha was increased to 0.10, but it did marginally covary with activity in the right STS at lags of 204 ms (peak *Z* = 2.904, *Z_crit_* = 3.14) and 230 ms (peak *Z* = 2.916, *Z_crit_* = 3.27). Critically, the backward cross-correlation showed no reliable association with DLPFC at lags prior to the seed activity even with an alpha of 0.10, indicating that the activity during the earlier reactivation does not have systematic associations with DLPFC.

The third and fourth seeds were taken from the peak activity observed at 742 ms for all trials and correct trials only, respectively. For the seed corresponding to all trials, we found cross-correlations with DLPFC both before (marginal at -153 ms, peak *Z* = 3.038, *Z_crit_* = 3.20) and after (at 102 ms, peak *Z* = 3.075, *Z_crit_* = 3.28) the STS activity (**Figure 6B**). The same pattern was found when examining the correct trials only. Activity in the DLPFC was correlated with the correct trials STS at a lag of -179 ms (peak *Z* = 3.385, *Z_crit_* = 3.16), and, marginally at a lag of 332 ms (peak *Z* = 3.039, *Z_crit_* = 3.14) and 358 ms (peak *Z* = 2.901, *Z_crit_* = 3.03; **Figure 6C**). This indicates that DLPFC activity systematically precedes and follows activity in the STS occurring around 700 ms.

## DISCUSSION

The goal of the current experiment was to reveal the spatial and temporal dynamics of cortical components of reactivation of relational memories by examining known face processing areas during the presentation of scenes associated with faces during prior learning. As predicted, we showed activation of face-sensitive cortex (the STS) not only to faces during study but also to associated scenes in the absence of face stimuli during test. These results are consistent with the interpretation that relational memories can reactivate stimulus-specific cortical areas in the absence of that stimulus type.

In order to establish that activation of face processing areas to scene stimuli in our face-scene relational memory paradigm was face-specific reactivation, we first had to identify one or more areas of the brain that were active for faces but not for scenes. In the study phase we found bilateral activation in the STS with activity greater for faces than for scenes in the left hemisphere around 400 ms and activity in the right hemisphere around 600 ms. The activity found in the STS is in line with previous research demonstrating that the STS is part of the face processing network (Puce et al., 1998, 2003; Grill-Spector et al., 2004; Fairhall and Ishai, 2007; Gratton et al., 2013). The latencies of both activities correspond to previous reports based on intracranial cortical evoked potentials (Allison et al., 1999), which also identified activity in the superior middle temporal cortex specific to face processing. Unlike previous work (Allison et al., 1999) we were unable to find activity in either side of the STS earlier than 400 ms. This could be due to a power issue generated by differences in signal-to-noise ratio between intracranial encephalography and EROS.

With the activity at study serving as a reference point, we found activation in the same and adjacent regions of the left STS to the presentation of scenes that were previously associated with faces. This relational reactivation took place in the left STS at two different latencies (around 500 ms and around 730 ms) with different patterns of activation for each time window. Notably, activation in the STS to the presented scene occurred only 100 ms later than activity elicited during initial study, demonstrating rapid reactivation of encoded faces to the presentation of relationally-bound scenes. The second reactivation occurred 330 ms later than activity elicited during study, and the pattern of activity was more anterior than that seen in earlier reactivation. Using a conjunction analysis, we found that there was overlap between the second reactivation and activity observed during the initial processing of faces, demonstrating that what we observed occurs in similar parts of STS. Conversely, we did not find any overlap between the face-related STS activity at study and the earlier STS activity during the scene preview: the early STS activity during scene preview was even more posterior than the late one and showed a different spatial configuration. This area of the STS has also been identified in previous studies as being part of face processing (Puce et al., 1998, 2003; Grill-Spector et al., 2004; Fairhall and Ishai, 2007) but was not identified during our study phase as being more active for faces than for scenes.

It is important to note, however, it should not be assumed that the activity observed in STS during the scene preview constitutes the entirety of the reactivated face representation. Just as there is a distributed network of processors that are active when viewing a face, likewise we assume that the reactivation is distributed across the face network. The activity observed in the STS only indicates that relational reactivation is taking place. Interestingly, recent evidence based on multi-voxel pattern analysis of fMRI data suggests that the STS is one of the areas holding representations of specific individual faces (Gratton et al., 2013). Future experiments could examine other face processing regions to gain a better understanding of the specific processes involved in episodic memory reactivation.

The contrast between the two different STS reactivations was further elucidated by the functional connectivity (cross-correlation) analyses between these voxels and the DLPFC. The early reactivation in the STS, characterized by the relatively small difference in latency between it and the activity elicited by faces at study, was systematically followed but not preceded by activation in the left DLPFC. The combination of the rapid reactivation and the lack of prior DLPFC activity suggest an automatic reactivation of face areas in response to the presence of relationally associated scenes. As the activity during the earlier time period is not associated with subsequent behavioral accuracy nor is it associated with areas of the prefrontal cortex (PFC), it is possible that this activity represents more the process or effort of retrieval and not the specific item that can later be used for the behavioral response. Conversely, the latter STS activation was both preceded and followed by activation in the left DLPFC, and was predictive of successful memory retrieval. Its later latency, coupled with the DLPFC activity that systematically precedes it, would indicate that the later period of activity may represent a top-down-driven retrieval process. This is consistent with the idea that a role of DLPFC is to maintain information necessary for an upcoming response, bridging a delay, as has been shown in delayed response paradigms (Fuster and Alexander, 1971; D’Esposito and Postle, 1999; Rowe et al., 2000; Rowe and Passingham, 2001). As the scene preview is essentially a preparatory period prior to the actual test display, one would expect DLPFC activation in conjunction with the active retrieval and maintenance of the successfully retrieved face in preparation for the upcoming response. More investigations involving single pulse transcranial magnetic stimulation of DLPFC right before or after the expected activation of STS may provide some more definite information about the causal relationships between the DLPFC and STS activities.

The interpretation of DLPFC activity as an index of the engagement of attention control mechanisms within the context of retrieval processes is also supported by the observation that this area appears to be active at multiple times during the preview period, in a manner interleaved with that of STS activation. In addition to the DLPFC activity we observed during the functional connectivity analyses, we also found activity in the ROI analyses in the left DLPFC early on during the scene preview (around 307 ms) and several instances of activity bilaterally in the DLPFC later on in the scene preview (from 1254 to 1715 ms). These findings are in line with Hannula and Ranganath (2009), who reported DLPFC activity during the scene preview, as well as numerous other studies investigating brain activity associated with the encoding and recall of relational items (Dobbins et al., 2002; Cabeza et al., 2003; Lepage et al., 2003; Duzel et al., 2004; Murray and Ranganath, 2007). As this is the first study to examine reactivation with this sort of temporal and spatial precision, it is difficult to compare the timing of the multiple observed DLPFC activations to the existing research. Previous event-related potential (ERP) studies have found sustained frontal positivities during the window of 300–1400 ms (Allan et al., 1996; Allan and Rugg, 1997) and others have found frontal activity from 600–2000 ms (Johnson et al., 1998). It is difficult to say whether the generators of these positivities are within the DLPFC. However, the broad spectrum of time in which these activites take place corresponds with the numerous instances of DLPFC activity that we observed. Further, the scalp distribution of the ERP positivities is consistent with generators located in DLPFC.

The laterality of activity in STS in the current study also may be of interest. During study, faces elicited activity both in the left and right STS. The right STS activation is consistent with previous findings (Kanwisher et al., 1997; Puce et al., 1998, 2003; Hoffman and Haxby, 2000; Ishai et al., 2000; Grill-Spector et al., 2004; Fairhall and Ishai, 2007). However, in the current study face reactivation response to relationally-bound scenes during the scene preview was only observed in the left STS. There was only marginal reactivation in the right STS, observable during the later reactivation period. It should be noted that in most studies of the involvement of STS in face processing participants were asked to passively view, or answer simple questions about, faces (Kanwisher et al., 1997; Puce et al., 1998, 2003; Hoffman and Haxby, 2000; Grill-Spector et al., 2004; Fairhall and Ishai, 2007) whereas the current study involved the retrieval, or reactivation, of faces in response to associated scenes. It is entirely possible that STS involvement in face processing is lateralized, with right STS more active than left STS in the processing of face stimuli when they are actually presented but not when they are only recalled. The laterality may then reflect the lateralization of the retrieval process rather than the lateralization of the face-related processes. The laterality of STS in various aspects of face processing will need to be more fully explored in future research.

A potential alternative interpretation for the STS activity during scene preview is that it may reflect a response elicited by the repetition of scenes. In fact, as we contrasted scenes that were previously paired with faces with novel scenes, it is theoretically possible that any observed activity during the scene preview could be due to greater STS activity for repeated scenes as compared to novel scenes. However the STS did not show increases in activity to the initial presentations of the scenes during either the initial study or during the presentation of novel scenes during test. It therefore appears unlikely that an area that is inactive during the initial presentation of a stimulus suddenly becomes activated upon the second presentation of that same stimulus. Whereas we cannot rule out this interpretation, it is far more likely that activity in the STS during the scene preview is due to face-related activity, an interpretation corroborated by research using other methods (Kanwisher et al., 1997; Puce et al., 1998, 2003; Hoffman and Haxby, 2000; Grill-Spector et al., 2004; Fairhall and Ishai, 2007).

Within the left STS, there may also be meaningful differences between the encoding of faces, occurring more anteriorly, and the retrieval of faces, occurring more posteriorly. The activity observed during initial study, elicited by face stimuli, and the reactivation during the scene preview, elicited by relationally-bound scenes, showed strong spatial overlap, at least for the later scene preview period (although not for the earlier period), but this scene-preview-elicited activity was more posterior than during initial study. Future experiments might explore this issue more directly.

## CONCLUSION

The current investigation sought to document the spatial and temporal dynamics of cortical components of memory reactivation. We examined activity in cortical face processing areas elicited by the presentation of scenes that during previous learning were associated with specific faces. Using EROS we found that participants activated the left cortical area STS not only when viewing faces during the study phase (relative to when viewing scenes) but also during a scene preview period at test for scenes that had previously been associated with those faces (relative to novel scenes). The activation of stimulus-specific cortical regions based on learned relations and in the absence of stimuli of that type, provides evidence of specific cortical substrates of the reactivation of relational memories. Participants activated STS in two distinct time periods during the scene preview. The activity in the early portion of the scene preview period, occurring just 100 ms later during the scene preview than that seen during the initial study phase, and not associated with prior DLPFC activity, may reflect the automatic reactivation of the associated face representation. The later scene preview activity in STS was associated with prior DLPFC activity and was predictive of behavioral performance, indicating that it might reflect a top-down, intentional retrieval of associated face information.

## AUTHOR CONTRIBUTIONS

All authors contributed equally to this text.

## Conflict of Interest Statement

The authors declare that the research was conducted in the absence of any commercial or financial relationships that could be construed as a potential conflict of interest.
